# Implementing a new emergency department: a qualitative study of health professionals’ change responses and perceptions

**DOI:** 10.1186/s12913-022-07805-w

**Published:** 2022-04-05

**Authors:** Nina Thórný Stefánsdóttir, Per Nilsen, Mette Bendtz Lindstroem, Ove Andersen, Byron J. Powell, Tine Tjørnhøj-Thomsen, Jeanette Wassar Kirk

**Affiliations:** 1 Department of Clinical Research, Copenhagen University Hospital Hvidovre, 2650 Hvidovre, Denmark; 2 Department of Health, Medicine and Caring Sciences, Linköping University, 581 83 Linköping, Sweden; 3 Emergency Department, Copenhagen University Hospital Hvidovre, 2650 Hvidovre, Denmark; 4 Faculty of Health and Medical Sciences, Department of Clinical Medicine, University of Copenhagen, 2200 Copenhagen, Denmark; 5 Center for Mental Health Services Research, Brown School and School of Medicine, Washington University in St. Louis, St. Louis, MO USA; 6 Department of Health and Social Context, National Institute of Public Health, University of Southern Denmark, 1455 Copenhagen K, Denmark; 7 Department of Public Health, Nursing, Aarhus University, 8000 Aarhus C, Denmark

**Keywords:** Change responses, Implementation science, Health care organization, Emergency departments, Acute medical units, Organizational change, Resistance to change, Change readiness, Interviews

## Abstract

**Background:**

The aim of the study is two-fold. It explores how managers and key employees at the Emergency Department (ED) and specialist departments in a university hospital in the Capital Region of Denmark respond to the planned change to a new ED, and how they perceive the change involved in the implementation of the new ED. The study investigates what happens when health professionals are confronted with implementation of policy that changes their organization and everyday work lives. Few studies provide in-depth investigations of health professionals’ reactions to the implementation of new EDs, and particularly how they influence the implementation of a nationwide organizational change framed within a political strategy.

**Methods:**

The study used semi-structured individual interviews with 51 health professionals involved in implementation activities related to an organizational change of establishing a new ED with new patient pathways for acutely ill patients. The data was deductively analyzed using Leon Coetsee’s theoretical framework of change responses, but the analysis also allowed for a more inductive reading of the material.

**Results:**

Fourteen types of responses to establishing a new ED were identified and mapped onto six of the seven overall change responses in Coetsee’s framework. The participants perceived the change as particularly three changes. Firstly, they wished to create the best possible acute patient pathway in relation to their specialty. Whether the planned new ED would redeem this was disputed. Secondly, participants perceived the change as relocation to a new building, which both posed potentials and worries. Thirdly, both hopeful and frustrated statements were given about the newly established medical specialty of emergency medicine (EM), which was connected to the success of the new ED.

**Conclusions:**

The study showcases how implementation processes within health care are not straightforward and that it is not only the content of the implementation that determines the success of the implementation and its outcomes but also how these are perceived by managers and employees responsible for the process and their context. In this way, managers must recognize that it cannot be pre-determined how implementation will proceed, which necessitates fluid implementation plans and demands implementation managements skills.

## Background

Emergency departments (EDs) are a primary entry point of about one million of the 1.3 million hospitalizations in Denmark [1] and a crucial part of the acute health care system, providing care for patients with acute injuries and illnesses. Emergency services in Denmark have changed over the past decade due to policy reforms by the Danish Health Authority [2] to implement new types of EDs (in Danish “Fælles Akutmodtagelser” or simply “FAM”). The initial background for this change was to diminish the risk of patients being admitted to a wrong “silo” of highly specialized physicians increasing the risk of wrong or missed diagnoses in the first hours of acute hospitalization [3, 4]. The ambition is to increase the efficiency and quality of emergency services and improve patient pathways in hospitals. Before the policy reform, EDs were primarily staffed with trainee physicians with limited access to supervision [5], and emergency care was often provided according to specialty at different units dispersed throughout different locations in the hospitals [2, 6, 7].

The reform designated 21 hospitals as emergency hospitals housing the new type of EDs which was a reduction from the previously 40+ hospitals with EDs [2]. The five Danish regions, who are tasked with ensuring the quality of the Danish health care system, were assigned the responsibility of implementing the recommendations in the reform, and the boards of directors of the hospitals had the responsibility of implementation within their respective hospitals, allowing for managers to influence the interpretation in the more detailed planning. The centralization of emergency services was expected to improve access to specialized facilities and equipment, as well as to multidisciplinary teams and senior physicians. Since 2007 the remaining emergency hospitals in Denmark have worked to implement new EDs in existing or new buildings [3]. The new EDs offer a single point of hospital entry for all emergency care patients (with some local exceptions such as children, women in labor, and citizens with psychiatric diseases [3]), 24/7 access to diagnostic facilities (e.g., laboratory and radiology, for effective emergency diagnostics and treatment), as well as continuous presence of senior physicians [8].

Similar reforms have been introduced in other countries to secure safe and efficient patient pathways for patients in need of emergency care [9]. The organizational structure of the new Danish EDs resembles so-called Acute Medical Units (AMUs) treating admitted patients for up to 48 hours before discharge to home or specialist department. The AMU model has been adopted in the UK, Australia and several European countries. However, the evidence base relating to the effect of the Danish ED model on quality of care is limited [10] and the evidence of the effect of AMUs on in-hospital mortality, mortality, and readmission rates is inconsistent [11–13]. Some international studies of the effect of similar ED configurations have investigated different aspects of the organizational intervention, e.g., the effect of centralization (see [14]), multidisciplinary teams (see [15]), and involving senior physicians or flow coordinators in patient triage (see [16, 17]). An overall interpretation of the effects of the organization of emergency medical services is complicated, as study populations vary, effect measures are narrow, settings are dynamic, and often more than one intervention is tested at the same time [18].

The process of establishing new EDs in Denmark has generated conflicts and debate about matters of allocation of responsibilities, criteria for preadmission assessment, professional skills, and concerns of reduced quality of care [19, 20]. Organizational changes, such as establishing new EDs, are often characterized by employees’ uncertainty and anxiety regarding how the change will affect their work lives [21]. For example, studies have shown that mergers and acquisitions often have an emotional impact on the involved managers and employees resulting in reactions such as anger, fear, and purposelessness [22–24]. Additionally, strategic, structural, and work-related uncertainty during a change can contribute to work-related stress and insufficient control of roles and tasks [25, 26]. It has long been argued that substantial change is not possible in health care without the engagement of health professionals [27].

It is of great importance to examine how health professionals, collectively and individually, experience, perceive, and respond to planned change, because it may be perceived to threaten positions and thus prompt negative reactions. Change responses play a significant role in orienting practioners’ decisions and behaviors, which influence implementation outcomes [28]. However, in implementation science, change responses remain unexplored both theoretically and empirically [29], and little is known of how local context shapes implementation processes and outcomes [30]. No previous studies have investigated health professionals’ responses to establishing new EDs in Denmark. Knowledge about health professionals’ change responses to this organizational change is key in identifying opportunities for promoting acceptance and limiting resistance to new ways of organizing and implementing EDs.

The aim of the study is two-fold as it seeks to explore how managers and key employees at the ED and specialist departments in a university hospital in the Capital Region of Denmark responded to the planned change to a new ED and how they perceived the change involved in the implementation of the new ED. Key employees are employees appointed by managers to play a central role in the implementation of the new ED.

### Theoretical framework

This study is grounded in Leon Coetsee’s theoretical framework of change responses [31]. Most conceptual work on attitudes and responses to change has been done on either readiness and acceptance to change or resistance to change [28] and the two concepts often appear in combination representing two opposite poles of a change response continuum [32]. Coetsee’s concept of change responses is conceptualized as a tridimensional attitude towards change: affective, cognitive, and conative (i.e., intentional-behavioral) reactions that may have implications for change. These three dimensions were introduced by early attitude theorists and have remained dominant within research on resistance to change [33]. The affective dimension is the feelings about change; the cognitive dimension relates to the opinion one has about the advantages, disadvantages, and usefulness of change; and the conative dimension concerns actions already taken or which will be taken for or against change [34].

Coetsee’s framework (Table 1) consists of seven forms of change responses on a continuum from commitment to aggressive resistance at each end of the continuum. Commitment is the most powerful acceptance of change, which requires employee empowerment. Involvement is a strong form of acceptance of change, which is demonstrated by taking part in the change by means of cooperation and participative behavior. Support is displayed through positive views on change although one does not necessarily act to promote or participate in it. Indifference is at the midpoint of the framework characterized by neutral attitudes and passive resignation to change. Passive resistance is a mild opposition to change (e.g., voicing negative views and considering quitting one’s job). Active resistance is a strong opposition to change, which involves negative attitudes and impeding behaviors (e.g., protesting). Lastly, Aggressive resistance is the most extreme form of opposition to change, which may involve efforts to prevent change (e.g., by means of spreading rumors, strikes, and even sabotage).

**Table 1:** Leon Coetsee’s framework of change responses [31]

	**Forms of response**	**Description**
	Commitment	The most powerful acceptance of change, which requires employee empowerment and acceptance of values and goals for achieving the organization’s mission.
	Involvement	A strong form of acceptance of change, which is demonstrated by taking part in the change by means of cooperation and participative behavior.
	Support	Displayed through positive views on change although one does not necessarily act to promote or participate in it.
	Indifference	The midpoint of the framework is characterized by neutral attitudes and passive resignation to change. Also described as the fourth form of resistance to change.
	Passive resistance	A mild opposition to change (e.g., voicing negative views and considering quitting one’s job).
	Active resistance	A strong opposition to change, which involves negative attitudes and impeding behaviors (e.g., protesting).
	Aggressive resistance	The most extreme form of opposition to change, which may involve efforts to prevent change (e.g., by means of spreading rumors, strikes, and even sabotage)*.*

Coetsee argues that acceptance and rejection of change should not be treated as separate and unrelated phenomena. Instead, the link between the two oppositions allows for a more complex analysis and view on the nature of responses to change, and as will become evident in this article, reactions to change are seldom black or white.

## Methods

### Study design and setting

This article is based on a qualitative study using semi-structured individual interviews. Semi-structured interviews enable the interviewer to push the conversation forward subtly, so prepared questions are covered while also allowing for pursuing interesting topics that arise during the interviews [35]. The interviews were conducted with managers and key employees employed at an urban emergency hospital in the Capital Region of Denmark. In Denmark, the health care system is publicly funded by taxes and the Danish welfare state provides free treatment for all citizens requiring medical care. The hospital in the study has around 700 beds, 5000 employees, 100,000 admissions a year, and a catchment area of +500,000 citizens. The board of directors constitutes the hospital top management and is supported by 18 clinical department managements in charge of clinical, financial, and organizational decisions within their departments. The ED has a bed section with 26 beds and an accident and emergency ward. The ED receives approximately 200-250 patients a day where 55 patients are admitted to the ED bed-section. This comprises a majority (70%) of all hospitalized patients, and the mean length of ED hospitalization is 13.2 hours before patients are transferred to specialist departments or discharged to home.

The organizational change of establishing the new ED broadly encompasses the following types of change: A merger of one part of a specialist emergency department (Department of Gastroenterology) with the current ED; a change entailing several specialist departments’ provision of beds and physicians to the new ED; relocation to a new building with new facilities and layout (e.g., single patient rooms only); and new ways of working, collaborating and organizing the ED. The number of beds in the ED bed-section will increase from 26 to 92 beds with up to 48 hours stays, and a short stays unit with up to six hours stays will be introduced. The ED has approximately 200 employees, which is expected to increase to 275.

The study forms part of a larger implementation research program initiated in March 2019 and expected to continue until 2023, when the new ED opens. The program uses multi-sited ethnography [36]; interviews and observations of local management meetings in the current ED; feature days about the new hospital; and so-called oilcloth sessions (in Danish “voksdug”). This is a micro-simulation method where managers, the board of directors, and health professionals work together on a blueprint with plastic figures representing ED staff on a scale of 1:50 to generate knowledge and workplace learning about the planned implementation. The research program is structured after Meyers et al.’s [37] Quality Implementation Framework (QIF), which serves as a conceptual overview of the steps of implementation of the new ED. The steps comprise four phases: Initial Considerations Regarding the Host Setting, Creating a Structure for Implementation, Ongoing Structure Once Implementation Begins, and Improving Future Applications. This study of change responses concentrates on pre-implementation and is part of the first phase, Initial Considerations Regarding the Host Setting, which focuses on the host setting and activities involving assessments of organizational needs, innovation-organizational fit, and capacity or readiness assessment. Meyers et al. point out that steps should be taken to foster a supportive climate for implementation and acceptance from key leaders and frontline staff in the organization [37].

### Participants

Our sampling strategy was inspired by the concept of information power [38]. This implies that the more information a sample holds, the lower N is needed. Five items impact the power of the sample, of which three are illustrated here. First, the sample size is dependent on the broadness of the study aim. To cover different aspects of the change process, the inclusion of participants from all involved departments was deemed necessary. Second, the specificity of experiences, knowledge, or properties among participants further relates to information power. Participants in our study had all participated in the oilcloth sessions. These were middle-level managers as well as key employees, designated by the board of directors and chief managers of the participating clinical departments, who were also present. The participants held different positions – some chief managers chose to invite other managers, such as middle-managers only, whereas others chose to invite nurses or trainee doctors. Finally, information power also relates to the analysis strategy of the study. In this study, we wished to perform a deductive analysis based on Coetsee’s [31] different kinds of responses. Thus, we invited everyone, who participated in the oilcloth sessions for an interview to secure a variety of participants, preferably with different positions within the organization. Participants were recruited via Microsoft Outlook calendar invitations. All in all, 62 persons were invited to participate, and 11 rejected the invitations. Rejections were typically given because of a heavy workload. Some did not answer the invitation. Receiving no answer, we sent reminder-e-mails and approached the person in question personally (based on personal relationship), and if an answer was not received or the invitation declined, the person was excluded from the study.

In total, we conducted interviews with 51 health professionals, who were employed in 12 different departments (Table 2). Participants were 26 physicians (10 chief physicians, 13 senior physicians, and three trainee physicians), 19 registered nurses (eight head nurses, eight charge nurses, an assistant charge nurse, a clinical nurse specialist, and one registered nurse), one head midwife, two managing medical secretaries, as well as one medical laboratory technician, one chief medical laboratory technician, and a head radiographer. To ensure anonymity, participants are presented as representatives of their specialty within the following four categories: emergency, medical, surgical, and other specialty, and not according to their position and profession.

**Table 2:** Participating departments

**Specialty**	**Department**
Medical specialty	Department of Cardiology Department of Gastroenterology (medical) Department of Infectious Diseases Department of Internal Medicine (including Department of Respiratory Medicine and Department of Endocrinology)
Surgical specialty	Department of Orthopedic Surgery Department of Gastroenterology (surgical)
Emergency specialty	Emergency Department
Other	Department of Clinical Biochemistry Department of Obstetrics and Gynecology Department of Pediatrics and Adolescence Medicine Department of Radiology

### Interviews

The semi-structured interviews were conducted between October 2019 and December 2020 by NS and JK. The interview guide was based on Coetsee’s [31] theory of change responses and covered different themes (Table 3). It was developed by NS and JK and revised by the rest of the authors. NS and JK pilot tested the interview guide with a senior consultant employed at the management secretariat of the hospital, which led to minor revisions. Interviews were conducted in meeting rooms in the hospital or the participants’ offices. All interviews were recorded and transcribed verbatim by a research assistant, resulting in 763 single-spaced pages. On average the interviews lasted 39 minutes.

**Table 3:** Interview guide

**Themes**	**Questions**
Introduction Thank you for your participation, written and oral consent Introduction of the research project and purpose Information about anonymity, confidentiality, recording, structure, and duration of the interview	
Introduction – About you	What is your job title and for how long have you been employed at the hospital? What is your role in relation to the establishment of the new ED?
Experiences with organizational changes	What are your experiences with other changes in your professional life? What kind of changes has succeeded? And why do you think they succeeded? What kind of changes has not succeeded? What went wrong?
The forthcoming implementation of the new ED	How do your previous experiences with change differ from the change you are facing with the establishment of the new ED? How would you describe your general attitude towards the new ED?
Preparation of the implementation	Please describe your thoughts and considerations in connection with the establishment of the new ED. What are the biggest benefits of establishing the new ED? (What are you looking forward to?) What do you see as the biggest organizational disadvantages of establishing the new ED? What do you get out of the new ED? What must you relinquish? What are your considerations on the physical framework of the new ED? (Worries and benefits) Are there certain physical conditions (e.g. rooms, appliances, etc.) that you find particularly important to be present in the new ED? Are there certain social conditions (e.g. events or activities) that you find particularly important to be present in the new ED? *Optional:* How will the ED influence your workflows and ways of working together/interacting? What did you think when you first heard about the new ED?
Opinions about the process of implementing a new ED	Do you experience a predominantly negative or positive attitude towards the upcoming ED among your colleagues? (How is it expressed?) In your experience, what are the employees occupied with in connection to the establishment of the new ED? (What kind of questions do they ask? And what do you answer them?) What stories are told in your department when the new ED is discussed (among employees and managers)? (Do you find that attitudes to (or reactions) vary according to (or are related to) the employees' professional background?)
Ideal conditions for change	What does it take for the new ED to succeed? What do you need? What do you want to do to make the transition to the new ED as good as possible for yourself and your co-workers? Do you do anything to convince your employees/colleagues that the new ED is a good/bad thing? If so - what do you do? And why?
Communication and information about the implementation of the new ED	How do you experience the atmosphere when the new ED is discussed with representatives from other departments at the hospital? (*Optional:* What words do people use about the process?) Who has the mandate in your department to make final decisions regarding the establishment of the new ED? (In relation todesign, organization, etc.) (*Optional:* Who do you think should have the mandate to make the final decisions regarding the establishment and organization of the new ED?) To what extent do you and your management team feel that you have an influence on the establishment of the new ED? Do you feel involved in the process? In what ways? How would you like to be involved? Is it your experience that there are decisions regarding the new ED that you are not involved in, but where you wish to be involved? How can you leave your mark on the new ED?
Rounding off and thanks	Do you have something on your mind? (Anything you think we need to know?)

### Data analysis

Analysis of the data was initiated when all interviews had been conducted and transcribed. NS carefully read each transcript to get a sense of the data set. Then quotes that could be categorized as a change response were placed deductively in a coding scheme in Microsoft Excel constructed for analysis. This was inspired by Coetsee’s [31] categories of change responses and also contained a section for other themes related to the change emerging from the material. Thus, we also allowed for an inductive reading of the material. The coding scheme initially consisted of seven columns (Table 4).

**Table 4:** Example of coding scheme

**Overall change response: Indifference**				
***Quote***	***ID no.***	***Position and department***	***Condensed meaning unit***	***Code(s)***
“[…] I just had a meeting with one of the deputy managing directors and all of the senior physicians to talk about his perspective on it [the new ED] and how to do it. How it is going to be and talk about it as it is a sort of condition that we cannot really discuss. This is the way it goes in the entire region and Denmark, so sure we can talk about it, but we can probably not change it.”	10	A representative from a medical specialty	We talk about the new ED, and I have attended meetings with the board of directors. The new ED is a condition, we can discuss but not change.	Indifference; the terms of the new EDs are final; surrender; top-down decision

The quotes were condensed into meaning units that were abstracted and labeled with codes [39]. To strengthen the validity of the analysis, JK read the coding scheme through when half of the interviews had been coded to reach an agreement on the codes. Finally, codes were clustered into subthemes within each identified change response. Simultaneously some subthemes were sorted out as they did not necessarily entail responses to the change, but rather characterized certain central aspects of the change, the participants reacted to. These were three overall themes, that were a result of the more inductive reading of the interviews.

## Results

In the first part of the results, the analysis of the participants’ change responses is presented and in part two, the participants’ perceptions of the new ED are presented.

### Change responses

Guided by Coetsee’s [31] continuum of change responses, the analysis resulted in 14 change responses (presented as (a) to (n)) mapped onto six types of overall responses to the implementation of the new ED (Table 5). The most extreme form of resistance, aggressive resistance, was not identified.

**Table 5:** Overview of participants’ change responses

**Forms of change response (Coetsee)**	**Categories identified in the material**
1. Commitment	(a) I feel a moral and ethical duty to promote the change (b) I believe in the value of the change
2. Involvement	(c) I work towards involving my medical specialty and profession in the change (d) My position in the organization obliges me to take part in the preparation for the change
3. Support	(e) I do not participate in the work towards the change, but I trust that the process is well-managed (f) I support the change because the new ED becomes a good learning environment (g) I support the change because I believe it will increase and improve the collaboration between the ED and specialist departments
4. Indifference	(h) I do not deal with the change (i) I believe that the change is a result of top-down decisions, which I cannot change (j) I feel ambivalent about the change
5. Passive resistance	(k) I am worried about the way the change is managed and conducted (l) I am worried about the outcome of the change
6. Active resistance	(m) I actively utter critique because the implementation process is not transparent and properly conducted (n) I do not believe that the change will bring about improvement
7. Aggressive resistance	Not detected in the material

### Commitment

The most powerful acceptance of the establishment of the new ED (i.e., commitment) was evident among a few of the participants, and these were representatives from the current ED. They provided statements that indicated commitment in two ways. Participants explained that they felt a moral and ethical duty to promote the change (a). A representative from the emergency specialty expressed:

“I must be able to get up in the morning and look at myself in the mirror and say: ‘You know what? What you are doing is the right thing’ guided by a moral slash ethical slash human compass.”

Participants expressing commitment often described an action-oriented behavior for ensuring successful implementation and fulfilling their duty. They intended to “set the agenda” and influence the board of directors. It was further perceived as a vote of confidence that the current ED management had been granted the management of the new ED by the hospital’s board of directors.

This acknowledgment made them further committed to ensuring a successful implementation of the new ED, and hence, this form of change response was characterized by a feeling of responsibility and recognition, which is characteristic of commitment according to Coetsee [31]. The establishment of the new ED was even described as one participant’s “heart’s blood”, and some explained that they as representatives of the ED were more committed than the specialist departments. Thus, collaboration with and gaining confidence from the specialist departments were crucial. On a cognitive level, participants expressed that they believed in the value of the change (b) by voicing their commitment to the general cause of changing Danish EDs. The change was experienced as a historical moment, and when motivating their staff, they invoked that the change was part of a greater transformation:

“We prepare them [the staff] all the time by telling them: ‘Listen, this will be the biggest change of the health care system in the next 40-50 years. You can influence it and show your initiative.”

This belief was further manifested in that the new ED could create better patient pathways as well as a better working environment for staff. In Coetsee’s understanding, commitment thus also entailed using and directing energy and loyalty for the benefit of the organization’s values and purposes [31].

### Involvement

Participants also showed involvement in the implementation process as they expressed how they worked towards involving their medical specialty and profession in the change (c). They sought to involve their staff bottom-up, though some of them experienced the change as being a result of top-down decisions. Participants often referred to earlier involvement in this process. For example, managers had been involved in the furnishing of different areas of the new ED (e.g., the laboratory facilities) and acknowledged that they had been involved earlier in the implementation process than in other hospitals undergoing the same change. Representatives from some specialist departments expressed that they considered themselves important actors in the current and future ED, and therefore they involved themselves in the implementation preparations. A representative from a medical department explained:

“The ED plays a very big role and it is a very big focus I have in my approach to being a manager of [my department]. [Our specialty] plays a big role in an acute hospital. And I take that very seriously. And I think we need a strong collaboration with the ED.”

This was further recognized by representatives from the emergency specialty, who expressed that they had an obligation to make the specialist departments “feel welcome”. However, they did not always feel that colleagues from the specialist departments showed interest in being involved in the implementation process. Additionally, some professional groups also showed involved behavior by directing attention to their profession. This was particularly evident among medical laboratory technicians and secretaries. A representative from the emergency specialty explained that she repeatedly drew attention to secretaries, so they were not forgotten in the change process - hoping that the management would not cut back on the secretaries once they were searching to find potential savings. Another aspect of being involved in the change included expressions of obligation, as participants explained that their position in the organization obliged them to take part in the preparation of the change (d). They considered change a natural part of their jobs as managers and health professionals with special areas of responsibility.

### Support

Supportive responses were articulated in different ways. According to Coetsee [31], support is characterized by the expression of positive views, without them being acted upon, meaning that in our case participants supported the new ED without working to promote it. Participants expressed that they did not participate in the work to promote the change, but they trusted that the process was well-managed (e). This meant that they trusted that the management of the current ED and the board of directors handled the implementation process professionally. Some expressed laissez-faire and a calm attitude and said they took the change in its stride as it was not immediately present. They trusted that the different aspects of the change would fall into their right places. A representative from a medical specialty felt that uncertainties were a natural part of implementation:

“One has learned to stay in the process, and on the way, things will fall into place, right? […] I have great confidence in the board of directors, that they have the complete overview, which I do not need to have.”

Positive utterances about the new ED concerned the new physical layout as well as the belief that patient care, treatment, and experiences would be enhanced in the new ED. Participants further mentioned that they supported the establishment of the new ED and the change it brought along because it became a good learning environment (f) - potentially offering a good learning environment and training processes across medical specialties, as medical specialties would now be physically closer to each other. Some managers also hoped that nursing staff was attracted to working in the new ED as it constituted a new career path. The changes were also supported because participants believed it would increase and improve the collaboration between the ED and the specialist departments (g). Wishes for future collaboration and a sense of community were expressed. It was believed that the new ED would “create a professional synergy“ between the specialist departments and the ED and that it would impact the “us and them” divisions that currently existed within the hospital.

### Indifference

Indifference was shown in different ways. Participants expressed that they did not deal with the change (h). The indifferent change responses had a temporal dimension since the question of how one felt about the pending change was constituted of earlier experiences as well as ideas of the future ED. Some experienced that the preparations and building process dragged out, some believed that nothing was settled yet and that the ED was “far out in the future” and yet others believed that the new ED was just a replication of earlier ways of organizing a hospital*.* A representative from a surgical specialty felt that the new ED was an old invention that had previously been abandoned because it did not work out. Representatives from specialist departments with special arrangements that exempted them from being fully integrated into the new ED, were not concerned about the future ED. They accepted the new ED, and it did not take up much of their energy. Participants were also “playing a waiting a game”, as they were awaiting further directions to be given on how the future ED would be organized. Additionally, participants described that they believed that the change was a result of top-down decisions, which they could not change (i). A representative from a medical specialty expressed:

“This is the way it goes in the entire region and Denmark, so sure we can talk about it, but we can probably not change it.”

In Coetsee’s framework, the indifference category is considered a zone between acceptance and rejection of change, and in our data, this particularly became evident when participants expressed indifference as a feeling of ambivalence about the change (j), because they some days felt confident in the success of the new ED, while on other days they were concerned. Representatives from the specialist departments expressed that they did not think of the new ED, but rather their own departments. Additionally, a representative from a surgical specialty explained how he felt ambivalent when hosting meetings for senior physicians in his department, who had many questions:

“There are so many uncertainties […] so in terms of management, we do not know. We are really in […] limbo, because if you could just say: ‘that is the way it is going to be’, it would be much easier.”

Other kinds of ambivalence were related to the vague evidence of the new organizational structure. It was mentioned that the new ED might make logical sense, but strong evidence for it lacked.

### Passive resistance

A lot of the participants expressed passive resistance to the change. According to Coetsee [31], passive resistance is demonstrated by negative perceptions and attitudes voiced as opposing views and regressive behavior. Passive resistance emerged when participants worried about the way the change was managed and conducted (k). Participants experienced that agreements could be fluctuant prompting frustration because it was believed that decisions needed to be made. Few thought that the board of directors was not firm in their decision-making. A representative from a medical/surgical specialty very bluntly expressed:

“I would rather like to have a board of directors who actually made some decisions. In reality, I find them non-existent, and I can hardly perceive them as my bosses in this process because they seem like they do not have an opinion […] or in fact have the competencies to manage this process.”

Representatives from the department that had to merge with the ED were particularly frustrated that the implementation process was not communicated as a merger, and efforts of involvement were not always experienced as involving. Because of insecurities and loose ends, managers described that they could not inform their staff, although they wished to. The category of passive resistance also contained different worries about the outcome of the change (l). Most of the participants expressed some sort of worry that varied in character and seriousness. Participants worried that the specialized knowledge and practice in the specialist departments would be lost and “watered down”. Another form of concern was the change’s effect on the remaining hospital. Some felt that the establishment of a new ED, from which the majority of acutely admitted patients would be discharged in the future, was the end of their department, which they had built up over years. It was feared that the circulation of staff between the ED and their department could potentially “split up” the specialist departments, that resources would be taken from the specialist departments and impact staff recruitment and retention of staff if receiving patients demanding a heavier workload. Several participants worried that both specialized nursing staff and senior physicians would quit if the forecasted change became a reality. Cultural differences between the ED and specialist departments were mentioned as roots to worries of increased collaboration in the future. A representative from a medical specialty explained:

“Internal medicine physicians […] take care of the outpatient clinics, so we also have a culture of actually wanting to work on day duty and then go home […] Every time our presence in taking shifts is increased, it has consequences for other day functions […]”

Some participants worried about applying time as a quality measure. This related to a political goal requiring that patients were attended by a senior physician within 30 minutes [4] and the introduction of a time limit assigned the bed unit of the ED (up to 48 hours). According to some participants, assessing patients according to their expected length of stay did not make sense. Thus, passive resistance was made up by a critique of the implementation process as well as worries about the outcome of the new ED.

### Active resistance

A more active form of resistance also occurred in the data material. This was present when participants recounted how they actively uttered critique because they thought the implementation process was not transparent and properly conducted (m). Participants challenged decisions they did not agree with at meetings, voiced irritation, and found it respectless when it was not clearly formulated who could make decisions regarding the new ED. A representative from a medical/surgical specialty had experienced that decisions were made in which her department had not been involved. This regarded a draft for the sections of the new ED in which their medical specialty had to share beds with up to five other medical specialties. She explained:

“And then we had to say: ‘no way, we simply do not want that’. And we feared that it would leak out to the staff. If that were to become the rumor, there’s the devil to pay. That is a real concern, either that they quit […] or that it ends in failure.”

She explained how other hospitals in Denmark had “mixed it all”. From the very beginning, she and her co-managers had repeated: “Let us not make the same mistakes”. Regarding the decision-making, a senior physician from the same specialty said:

“[…] To me it would be good if they could announce who has the decision-making authority […] who decides because it would be […] more respectful. Instead, you have the feeling that there is an underlying agenda of which we hear nothing of.”

Participants also expressed that they did not believe that the change would bring about improvements (n) and expressed critique of the implementation process and the fundamental principles of the new ED, which they believed would not improve acute care. It was believed that it was not beneficial to break down the medical specialties, that it was ill-prioritized to save resources in the specialist departments to increase it in the ED, and the principle of continuous presence of senior physicians in the ED particularly attracted criticism. A representative from a surgical specialty explained that he found it hard to see how the costly resource of senior physicians was allocated to the ED when he could not see the point of their presence there, while the specialist department lacked resources. A representative from a medical specialty explained this critique:

“If one believes in breaking the professional competence by forcing the people to work somewhere because one has a political idea that it is a good idea, you will be in trouble.”

Thus, the strongest form of resistance to the establishment of the new ED among the participants was characterized by them actively uttering critique of the implementation process and the fundamental principles of the new ED.

### Perceptions of the new ED

Contextual factors inside and outside the organization influenced the ways the participants reacted to the forthcoming change. Therefore, this next section is dedicated to the results that emerged out of the inductive reading of the material when performing the deductive analysis. It answers the second part of the research aim of understanding how the participants perceived the change involved in the implementation of the new ED. The findings are structured in three themes that traversed the change responses and illustrated the participants’ perceptions of the forthcoming change - the change they reacted to (Table 6).

**Table 6:** Overview of participants’ perceptions of the new ED

**Perceptions of the new ED**	**Short description**
1. Changing patient pathways	Disagreements on what constitutes the best possible acute patient pathway and whether the planned organization of the new ED would redeem this
2. Changing the physical layout of the ED	Different opinions about the new ED’s location in a newly built wing of the hospital, which poses both potentials and challenges
3. A new medical specialty gaining its foothold	Hopeful and frustrated statements about the newly established medical specialty of EM, which is related to the implementation of the new ED

### Changing patient pathways

A general and not surprising pattern in the material was the participants’ expression of a common goal of establishing the best possible acute patient pathways. In the future, the organization intended to follow the course of the patients’ pathway with as few transitions as possible. One participant expressed that this was a “fundamental, huge change” in the way patient pathways were thought of. A representative from the emergency specialty pointed out that the establishment of the new ED was a question of a change in a physician culture where they determined the course of the patient. In the future, the organization would instead be determined by the patients and their demands, because senior physicians would serve as ED frontline staff: “It is no longer the physician, who determines the system, but the patient”. Some participants believed that the new ED changed patient pathways for the better, while others expressed worries that the quality of the patient pathways worsened. Often participants expressed hope that the new ED would shorten waiting times for patients, as the pathways were optimized, and transitions made fewer. This was linked to the diagnostics being placed “at the door” of the ED and because the physical surroundings were enhanced. Additionally, it was mentioned that frontloading in terms of senior physicians’ presence in the ED would benefit patients. Others thought that the increased presence of senior physicians was not necessarily deemed better for the patient, as this organizational model was not attractive to the involved physicians and caused an experience of loss of privileges for some specialist physicians. A representative from a surgical specialty described that trainee physicians with few exceptions were just as qualified to refer patients to the surgical departments as senior physicians. He explained:

“So, it may well be the case that patients get a more competent treatment, but it may well be that it gets less good because it is not in our interest to tend to minor injuries and such.”

Representatives from specialist departments also voiced worries that patients belonging to their specialty would be kept out of their department.

### Changing the physical layout of the ED

Another central aspect of the establishment of the new ED was its location in a newly built wing of the hospital. The spatial dimension of the change constituted a place imbued with prescribed meaning [40] and imagined futures. The physical space of the new ED spurred both enthusiasm and concerns. For some, the new building set the stage for re-thinking patient pathways and optimizing the working environment and collaboration between professions and medical specialties. But participants also expressed concerns about the new surroundings. It was mentioned that the design and plan arrangement did not make sense, as a representative from the emergency specialty explained:

“[…] the physical environment is not thought through in our world, but it may be thought through in an architect’s world.”

Several participants expressed worries about overview of the ED, lack of certain rooms or functions, such as conference rooms, staff facilities, disinfection rooms, pneumatic posts as well as big walking distances. Another aspect related to the new building was frustrations when participants had participated in the design process earlier but felt that their ideas were not reflected in the eventual outcome.

### A new medical specialty gaining its foothold

The third aspect characterizing the change responses to the new ED was the participants’ ways of relating to the establishment of the new emergency medicine (EM) specialty. Both hopeful and frustrated statements were uttered about it and this touched upon lack of recognition and specialty identities and hierarchies. The new specialty was established in 2017, as it was expected to increase quality and efficiency in the EDs [4]. Some participants recognized that the success of the new ED was dependent on the success of EM, and they voiced that representatives of the EM specialty lacked recognition and support from the specialist departments. A representative from the emergency specialty said:

“It is important that it is articulated throughout the organization […] that this is what we want, […] and that the emergency medicine specialty is here to stay.”

Participants with different professions, that is both physicians and nurses, from the ED, worked for the development and recognition of the EM specialty, though it yielded challenges. According to the participant quoted above, the well-established specialist departments had a “strong professional identity” whereas the new emergency specialty “was in the process […] of building itself up”. Several respondents mentioned that the EM specialty lacked acknowledgment. Participants from the ED experienced lack of trust and acceptance among their colleagues in the specialist departments and experienced criticism of their professional expertise, e.g., at meetings. This disapproval was visible in the condescending use of language used about EM physicians, which for example were termed as “radiator physicians”:

“Once physicians graduate from university, they are physicians. However, it will not take long before they are either endocrinologists, cardiologists […] and you name it […] and there will always be teasing across specialties. So, I am not sure whether it is this [teasing] or the lack of professional expertise that finds expression in the description of emergency medicine specialists as ‘radiator physicians’ […]. It comes from when you lean against a radiator, that you look at a screen and […] you do not do much more.”

Especially representatives with a physician background from the specialist departments mentioned that it would take years before enough EM physicians had been qualified, which caused challenges with recruitment. In these years, this gap instead had to be filled by physicians from specialist departments. It was however acknowledged that the specialty was in a process of establishing itself. A representative from a medical/surgical specialty explained the lack of status and prestige encompassing working in an ED:

“[…] It is not cool to be an emergency medicine physician […]. Maybe among emergency medicine physicians but other than that it is not too cool. And it is a limping specialty, not because we do not need it […] there is no formal education […] maybe in ten years it will be different.”

These three themes showcase the complex nature of the change of establishing a new ED, and thus the aspects of the change, that the participants responded to.

## Discussion

Implementation of a fundamental change of the Danish ED system is a complex matter, and the current study showcases the challenges and potentials of implementing policy with relatively vague guidelines for operationalization. Thorough work is needed when new practices – be they evidence-based or not – are introduced in complex health care settings [37]. This study aimed to explore how managers and key employees responded to the planned change to a new ED and how they perceived this change. On one level, the study demonstrated the multiple ways, managers and key employees respond to organizational change. Coetsee’s [31] change responses framework served as a useful categorization for understanding their responses to future change. We identified 14 types of change responses that were mapped onto six of the seven responses in Coetsee’s framework. On another level, the study provided insights into the ways the participants perceived the organizational change of establishing a new ED. Common for them was that they responded to three different aspects of the new ED. First, they all expressed a wish for creating the best possible acute patient pathways. Opinions as to whether the planned organization of the new ED would redeem this varied. Second, the participants responded to the fact that the new ED would be located in a new building, which both posed potentials and challenges. Third, both hopeful and frustrated statements were given about the newly established medical specialty of EM, which was related to the implementation of the new ED. This particularly confirms that it is not only the content of the implementation that influences the process or outcomes but rather the manifold ways this content is perceived and responded to as well as the context in which the implementation takes place.

Our study showed that not only did the health professionals react differently to the forthcoming change, they also sometimes expressed attitudes that contained both positive and negative aspects. As such, attitudes can fluctuate and change as they are related to context and temporality [41]. In our case, we studied a change that was about to be carried out (pre-implementation); and inquiring into change responses later in the process would possibly generate different results. The following sections are structured as a discussion of the three levels in Coetsee’s framework – that of change acceptance and readiness (commitment, involvement, and support), change indifference, and change resistance (passive, active, and aggressive resistance) – as they appear in our material and in relation to the three contextual circumstances, that stimulate them.

### Creating commitment, involvement, and support

Commitment was evident among a few of the participants. They felt a moral and ethical duty to promote the change and believed in the value of the change. They were actively engaged and involved in implementing the new ED and expressed willingness to use and direct their energy for the benefit of the new ED and wider organization. These actions were linked to a strong belief in the concept of the new ED and the EM specialty. However, this effort was not without challenges. On an everyday basis, they had to defend and vindicate the new ED organization and the EM specialty. The Danish Health Authority introduced the organizational change of the EDs in 2007, but it was not until 2017 EM was established as a new specialty. The introduction of the specialty was prolonged due to structural barriers and worries about the quality of acute care in the EDs [4, 42]. The EM specialty has long been an established medical specialty in Australasia, Canada, Ireland, the UK, and the US [43], but is a much newer idea in Europe [44]. In a Scandinavian context, Sweden has come the longest way in implementing EM [45], and in Norway and Iceland promoters of EM have similarly experienced resistance from established medical specialties, as in Denmark. Resistance to the establishment of a new medical specialty of EM was mainly raised by well-established medical specialties, who e.g. argued that other specialties such as anesthesiologists already competently managed critically ill patients and it was proposed that existing specialties should send attending physicians down to receiving areas to supervise [46, 47]. Skeptics’ resistance in Norway was overcome by using Zink’s [48] work on the history of EM in the US as a playbook and support for the specialty, as well as demonstrating the goal of doing the best for the patient to policymakers and the general public through the use of the media [47]. In Iceland, creating acceptance of the new specialty was aided by visiting international EM physicians, by getting medical students and graduates interested in EM, by formalizing educational and training programs, by establishing an EM society, and by hospitals hosting case conferences with other specialties for them to obtain better understanding of how the patient’s course was in the ED, what was done and why. Additionally, as the specialty became more established, research activities were increased [46]. Participants in our study who expressed commitment often referred to having the political wind at their back, as the fundamental decision of strengthening the acute area in Denmark, in the end, was a political decision. This, however, was not necessarily deemed convincing by specialist departments, who e.g. demanded evidence for the establishment of the new ED and its organization, rather than political argumentation. The same challenges were found by Pedersen et al. [20]. In their study, EM physicians experienced negative reactions from the specialist departments, who did not believe in the skills of the ED physicians and held that placing them up front in the new ED would impair patient treatment. This challenge with legitimacy was similarly voiced in our material, and at times an opposition between “us” and “them” was created, which posed a barrier to the implementation of the new ED. This points to the challenges of establishing a new medical specialty. Existing specialties often experience identity threats when confronted with a new specialty, as this development causes them to reposition their domain. This threat is mostly experienced by specialists with strong professional identities and may lead to implicit or explicit identity struggles between specialties [49]. On a more general note, change, or the prospect of it, is particularly likely to provoke concerns about identity [50]. Within different disciplines, it has been widely recognized that identity entails both individual and collective aspects [51]. In this way, identity is not just about individual understandings of it, but equally about statuses, roles, and social positions [50]. This means that communication about and voicing of identity are not enough, they must be accepted by others before the identity is “taken on”. The process of identification thus is to be found and negotiated at its boundaries. Thus, individual and collective identities are interactional products of external identification made by others as well as self-identification on an internal level. One’s identity must be validated by others [50]. These lines of thought can help explain why participants expressing commitment met challenges in relation to EM identity in their meetings with other, strong specialized identities. However, more extensive research on the challenges of hierarchies of medical specialties, specialty identities and the reluctance to accept new specialties is needed.

Participants who expressed involvement worked towards involving their medical specialty and profession in the change and said their position in the organization obliged them to take part in the preparation of the change. In this way, by virtue of their position as managers or key employees they felt a responsibility for conducting the necessary work for assisting and leading the approaching change. The relevance of leadership in implementation science has gradually been acknowledged and studies have underlined the importance of the role of leaders and managers in implementation processes [52]. Managers’ experience of the implementation process in health care and their effect on implementation outcomes are generally unknown [53]. At the pre-implementation stage in our study, managers and middle managers played a significant role. They expressed how they participated by virtue of their role as managers, and how they worked to influence the process, but leadership and managers’ importance in the early stages of implementation needs further investigation. Participants who voiced support, did so because they believed the new ED would become a good learning environment, that the collaboration between the ED and the specialist departments would increase and improve. However, they did not necessarily participate in the work to promote the change but trusted that the process was well-managed. This is interesting as it points to the other ways of managing implementation. What was at stake among participants who supported the implementation of the new ED, but did not work actively to promote it, related to their perception of their role in the implementation process.

### In-between – indifference and ambivalence

Some of the participants voicing indifference said that they did not deal with the change. They believed that it was a result of top-down decisions, they could not change and expressed feelings of ambivalence. Coetsee describes indifference as a “neutral or transition zone characterized by a lack of negative emotions or attitudes” [31]. Though the new ED might be considered a ground-breaking re-organization by some, participants expressing indifference did not necessarily experience it as such. This was partly related to a matter of temporality, which was evident in different ways. A lot of the participants expressed that the ED belonged to a far future, and they rather dealt with different current implementation projects and operations of their department. This resonates with research showing how implementation efforts can add significant staff burden, which can reduce quality of patient care and may even impact treatment efficacy if the interventions disrupt workflow [54]. In this way, participants expressing indifference, rather focused on their current departmental operation, than on the future organizational change. This shows how they were dealing with a change that was decided in the past but would be executed in a future they were perhaps not going to be part of, possibly leading to less ownership of the implementation process. Some departments had negotiated special agreements with the board of directors which exempted them from being present in the new ED, and yet others felt that the new ED was just a replication of earlier organizational configurations. This, in a way, resembled a sort of innovation or change fatigue. Chung et al. [55] define innovation fatigue as “the exhaustion of emotional and cognitive resources of an employee that disrupts his or her further engagement in subsequent innovations”. In our material, this was particularly visible when participants expressed that with the new ED being a top-down change they could not do much more than to follow along. Mandated top-down change has often been perceived as more difficult to implement than change that is based on a bottom-up perspective [56].

Additionally, indifference was characterized as a feeling of ambivalence. Repovš et al. [32] have suggested that researchers pay more attention to understanding the spectrum of ambivalence toward change, as individuals’ attitudes to change are rarely bipolar. Ambivalence in this study was constituted of feelings of uncertainty about the organization of the new ED as well as the existing hospital, which placed participants in limbo*.* Participants found themselves somewhere between being supportive while still being worried – between acceptance and rejection of change. This resembles the classical anthropological concept of liminality [57, 58] covering the experience of a stage of transition between a former well-known situation to an impending, often uncertain one. Facing a transition prompts questioning of who one is while facing the transition, but also who one will be in the future. Liminal processes can be fruitful but also unsettling and threatening, and in this way, the concept captures how participants in our study were both supporting and worried. The uncertainty characterizing liminality may provoke stress and anxiety [59].

### Resistance to the establishment of the new ED

The most prevalent change response detected in our material was passive resistance. Participants expressed worries about the outcome of the change and the way the change was managed and conducted. Worries were manifold, varied in character and seriousness, and both concerned the organization of the new ED as well as the remaining hospital. These voiced worries as well as stronger forms of resistance, such as active resistance, indicate the necessity of managers addressing them as well as acknowledging that resistance can be a resource for change [60]. The strongest form of resistance, active resistance, to the implementation of the new ED was evident among participants, who explained that they uttered critique, because they thought the implementation process was not transparent and properly conducted, and they did not believe that the change as it was presented to them would bring about improvements. With reference to Rogers’ [61] much-cited theory of diffusion of innovations, Stewart et al. [62] have called for a critical re-thinking and scrutinization of the category of laggards or non-adopters. They argue that these have much to offer researchers about attitudes to and viability of evidence-based practices in their settings. They may not be convinced by traditional implementation strategies but may be more palatable if strategies are designed as tools to be integrated into the ideology of helping the suffering. This is very relevant in relation to the participants who expressed active resistance in our study – especially those who showed the most resistance. Worries and resistance were based on ideas of wanting what was best for the patients. However, this was perceived differently, which complicated the implementation process. Participants also expressed discontent with the conduction of the change process. They felt that agreements constantly changed, that the board of directors did not listen, and they were frustrated that the change process was not voiced as a merger. Studies have shown that support from the organization is important for managers’ commitment to change and lack of support can have negative consequences [63, 64]. Our results showed that resistant participants were particularly prone to critiquing the board of directors and other managers for their ways of managing the change process. They questioned their communication style, the lack of clear lines, and the general guidelines for the new ED.

### Implications for clinical practice – managing in the winds of change

In modern organizations, such as large public hospitals, change is a constant. This study highlighted different aspects to be taken care of during implementation and thus has implications for clinical practice to be considered, especially in questions of managing implementation processes. Aarons et al. [52] have presented a model for leadership in implementation within health care. This includes four aspects that managers need to cover to achieve an effective implementation process. First, they must be proactive, produce and communicate a plan for the implementation, and address barriers to the implementation. Our findings suggest that communication plays a significant role in implementation efforts. Sometimes this was a matter of terminology, as participants expressing active resistance particularly demanded that the change process was articulated as a merger. It was believed that by not calling the implementation process a merger, the significant consequences a merger has to work environment and identity would be underplayed. When different departments are brought together and changes are made in management, staff composition, relationships, or procedures, prior understandings of identity are challenged; they disturb employees’ understandings of sameness and difference in relation to others [65], which can cause uncertainty and tensions. Participants expressed that they particularly struggled with communicating the change to their staff, when the final lines had not been drawn. Aarons et al. [52] also underline that managers must have knowledge and understanding of implementation issues and be able to answer staff questions about the implementation. In our material, this was sometimes not possible, and participants felt that they lacked the necessary skills to communicate things they were uncertain about, thus instead they held back information because they did not want to transfer their uncertainties to their employees. Additionally, the initiation of the ED depended on a new building being ready for occupation; a process that dragged on and to many was an abstract physical space. Aarons and colleagues [52] also point to the importance of managers appreciating employee implementation efforts, supporting and giving feedback. This also implies listening to resistant employees and working with them to share an understanding of the common problem they together are trying to fix. The findings in our material deliver insights to managers and hospital directors about specific aspects of worries or resistance that enable them to tailor implementation strategies that accommodate these. These are for example worries about loss of specialized professional competency; the change’s effect on the remaining hospital: cultural differences between the ED and specialist departments; specialized nursing staff and senior physicians quitting their jobs if the forecasted change became a reality; applying time as a quality measure; and the fact that the implementation process was not communicated as a merger. According to Aarons [52] managers must also be perseverant and reactive and thus constantly address challenges as they arise throughout the implementation process. Thus, our material shows the importance of an ongoing dialogue about how different actors in a system perceive the overarching purpose of implementation initiatives and the resources available to achieve that purpose. Further, awareness must be paid to those aspects that surround and influence the implementation process. This especially relates to the challenges of establishing the new EM specialty, which is deeply intertwined in both accepting and resistant views on the new ED. It must also be recognized how an “us and them culture” is put to the fore when changing a big organization such as a hospital. The policy framework set out by the Danish Health Authority presented an ambition of a strengthened collaboration once different departments and units were merged and centralized. However, our material points to an inherent culture of “us and them”, which is deeply rooted in the organization. This might fertilize conflicts and possibly lead to avoidance of contact and collaboration – a process that often reproduces conflicts. Senior physicians may be key in this, as this study has shown that the implementation process deeply touches upon specialty identities. Physician managers are carriers of their medical specialty mentality, which may become reproduced, and thus both present potentials and challenges to the construction of the new ED.

### Strengths and limitations

Some important limitations of the study should be noted when interpreting the findings. The study presents a single case as it is based on interviews with representatives from one hospital. Interviews with representatives from other hospitals’ re-organizing and establishing new EDs could have contributed with other relevant perspectives. However, the current study holds a relatively large sample for a qualitative study and thus gives an in-depth knowledge of the different change responses at play. Additionally, the study was conducted before the active implementation of the new ED and thus presents ideas and imaginations of what the future ED might come to look like, rather than focusing on the responses to the actual organizational change and implementation. A note should also be made on the fact that the selection of participants indirectly was made by the board of directors and chief managers of the participating departments, as they selected participants for the oilcloth sessions. In this way, the researchers did not include representatives from other departments, who could have been relevant, e.g., from the physiotherapist and occupational therapist department or the service department. However, the representatives interviewed in this study were those who were activated in the organization at this point of the implementation process, thus representing this real-life step of the implementation process, rather than our opinion of central actors as researchers. We were aware that the implementation of the new ED entailed various other change processes, anticipated as well as unanticipated, which we did not cover in this study (e.g., IT mergers and change in the provision of service functions.) Lastly, this study applies Coetsee’s [31] framework of change responses before the planned implementation of the ED, rather than in retrospect. Therefore, the results may be interpreted as an up-to-the-minute account in a context where things in the implementation process still needed to be settled and were unclear. This, however, may also be viewed as a strength of the study, as it shows the potential of Coetsee’s framework as a predictive tool, as the insights may also prompt the possibility of acting on the responses early on in the process. Future research could explore how to deal with these responses.

## Conclusions

In conclusion, this study has demonstrated the different ways managers and key employees at the ED and specialist departments in a university hospital in the Capital Region of Denmark responded to and perceived the planned implementation of a new ED. Coetsee’s [31] change responses framework served as a useful categorization for understanding their responses to future change. We identified 14 types of change responses that were mapped onto six of the seven responses in Coetsee’s framework. The participants perceived the change as particularly three changes. Firstly, they all expressed a wish for creating the best possible acute patient pathway in relation to their specialty. Opinions as to whether the planned organization of the new ED would redeem this varied. Secondly, the participants responded to the fact that the new ED would be located in a new building, which was both full of potential as well as worries. Thirdly, both hopeful and frustrated statements were given about the newly established medical specialty of EM, which was deeply connected to the success of the new ED. The study showcased how implementation processes within health care are not straightforward and that it is not only the content of the implementation that determines the success of the implementation and its outcomes but also how these parts are perceived by the managers and key employees responsible for the process, as well as the context they are surrounded by and with which they constantly interact. In this way, managers must keep in mind that it cannot be pre-determined how implementation will proceed, which necessitates a fluid implementation plan and demands implementation managements skills.

## Data Availability

The data set generated and analyzed during the current study is not publicly available as it contains potentially identifying or sensitive information that could compromise the privacy of the respondents according to regulations set out by the Danish Data Protection Agency but is available from the corresponding author on reasonable request.

## Abbreviations

AMU: Acute Medical Unit
ED: Emergency Department
EM: Emergency Medicine
UK: The United Kingdom
US: The United States
